# Clinicopathological Characteristics of Odontogenic Keratocysts in the Mexican Population

**DOI:** 10.3390/diagnostics16142194

**Published:** 2026-07-14

**Authors:** Karla J. Rios-Orta, Brenda J. Valdez-Vargas, Paola L. Acevedo-Quezada, Edith Lara-Carrillo, Evelyn V. Flores-Solano, Norma G. Ibáñez-Mancera, Montserrat Escuadra-Landeros, Jonatan E. Flores-Riveroll, Penélope I. Manzano-Galindo, Guillermo Molina-Vidal, Marcos Hernández-Nava, José E. Garduño-Mejía, Saray Aranda-Romo, Francisco J. Tejeda-Nava, Ana M. Santillán-Reyes, Sergio S. Utrera-López, Norma Samanta Romero-Castro, Salvador Reyes-Fernández, María F. Lara-Fonseca, Ulises Velázquez-Enriquez, Elías N. Salmerón-Valdés, Rebeca S. García-Toral, Adrián Juan Hernández-Cruz, Adriana A. Morales-Valenzuela, Oscar Ramos-Carrillo, Fátima Del Muro-Casas, Victor H. Toral-Rizo, Rogelio Gonzalez-Gonzalez

**Affiliations:** 1 School of Dentistry, Autonomous University of the State of Mexico, Toluca de Lerdo 50130, State of Mexico, Mexico; kariosor7717@uaemex.mx; 2 Orocentro Oral Medicine and Pathology Clinic, School of Dentistry, Autonomous University of the State of Mexico, Toluca de Lerdo 50130, State of Mexico, Mexico; bvaldezv001@profesor.uaemex.mx (B.J.V.-V.); elarac@uaemex.mx (E.L.-C.); vfloress@uaemex.mx (E.V.F.-S.); amsantillanr@uaemex.mx (A.M.S.-R.); ssutreral@uaemex.mx (S.S.U.-L.); uvelazqueze@uaemex.mx (U.V.-E.); ensalmeronv@uaemex.mx (E.N.S.-V.); aamoralesv@uaemex.mx (A.A.M.-V.); 3 School of Dentistry, Autonomous University of Baja California, Mexicali 21040, Baja California, Mexico; paola_lisset.acevedo@universidad-uic.edu.mx; 4 Interdisciplinary Health Sciences Center (CICS), Santo Tomás Unit (UST), Instituto Politécnico Nacional, Mexico City 11340, Mexico; nibanezm@ipn.mx; 5 Division of Health Sciences, School of Dentistry, Universidad Intercontinental, Mexico City 14420, Mexico; montserrat.escuadra@universidad-uic.edu.mx; 6 School of Dentistry, National Autonomous University of Mexico, Mexico City 04510, Mexico; 7 Puebla Maxillofacial Center, Puebla 72197, Puebla, Mexico; 8 Cholula General Hospital, San Andrés Cholula 72825, Puebla, Mexico; 9 Maxillofacial Surgery Service, “Licenciado Adolfo López Mateos” Medical Center, Toluca de Lerdo 50226, Estado de México, Mexico; markcnava@gmail.com (M.H.-N.); jgardunom@uaemex.mx (J.E.G.-M.); 10 School of Stomatology, Autonomous University of San Luis Potosi, San Luis Potosi 78290, S.L.P., Mexico; sarayaranda@fest.uaslp.mx (S.A.-R.); francisco.tejeda@uaslp.mx (F.J.T.-N.); 11 Academic Unit of Dentistry, Autonomous University of Guerrero, Acapulco 39070, Guerrero, Mexico; normaromero@uagro.mx (N.S.R.-C.); salvadorreyes@uagro.mx (S.R.-F.); 12 General Hospital of Acapulco, Guerrero State Health Secretariat, Acapulco 39901, Guerrero, Mexico; 13 Pediatric Dentistry, School of Dentistry, Autonomous University of the State of Mexico, Toluca de Lerdo 50130, State of Mexico, Mexico; mlaraf449@alumno.uaemex.mx; 14 Department of Health Sciences, Autonomous University of the State of Mexico, Toluca de Lerdo 50130, State of Mexico, Mexico; rgarciat006@alumno.uaemex.mx; 15 La Perla General Hospital, Nezahualcóyotl 57820, State of Mexico, Mexico; adrian_hdzc@hotmail.com; 16 Oral Surgery and Comprehensive Diagnosis and Oral Patology, Private Practice, 307 Venustiano Carranza Nte., Col. Santa Teresita, Tepic 63020, Nayarit, Mexico; oramosc1973@hotmail.com; 17 Academic Unit of Dentistry, Autonomous University of Zacatecas, Zacatecas 98160, Zacatecas, Mexico; fatima.delmurocasas@uaz.edu.mx; 18 Research Department, School of Dentistry, Juárez University of the State of Durango, Durango 34000, Durango, Mexico

**Keywords:** odontogenic cysts, histology, keratins, tomography, pathology

## Abstract

**Background/Objectives:** Our aim is to describe the clinical, imaging, and histopathological features of odontogenic keratocyst in a Mexican cohort. **Methods:** This is a retrospective, descriptive, and cross-sectional study. Clinical records and histopathology reports (2015–2025) from multiple laboratories were reviewed. Slides stained with hematoxylin and eosin were evaluated by pathologists using standardized criteria. Descriptive statistics were performed. **Results:** A total of 106 cases were included. Ages ranged from 10 to 86 years (mean 33.4 ± 17.7). There was a predominance of males (61/106; 57.5%). The most frequent location was the posterior mandible (49/106; 46.2%), followed by anterior mandible (30/106; 28.3%), posterior maxilla (19/106; 17.9%), and anterior maxilla (8/106; 7.5%). Most cases were asymptomatic (73/106; 68.9%). Association with an impacted tooth was documented in 37/106 (34.9%) and root resorption in 26/106 (24.5%). Satellite epithelial islands were observed in 8/106 (7.5%), satellite cysts in 17/106 (16.0%), and both findings in 4/106 (3.8%). Chronic inflammation was present in 62/106 (58.5%). **Conclusions:** In this Mexican series, odontogenic keratocyst predominated in males and, in the posterior mandible, was frequently asymptomatic, and showed satellite foci and chronic inflammation in a relevant proportion, reinforcing the need for clinical-imaging and histopathological correlation, as well as follow-up.

## 1. Introduction

Odontogenic keratocysts (OKCs) are benign intraosseous lesions that are slow-growing, asymptomatic, and have a high recurrence rate. They develop from remnants of the dental lamina and account for approximately 10% of maxillary cysts. The term odontogenic keratocyst was coined in 1956 [1] and, after being classified as a “keratocystic odontogenic tumor” in 2005 by the World Health Organization (WHO) [2], OKC was reclassified as a cyst in 2017 [3]. At present, the diagnostic criteria for head and neck tumors in the most recent edition of the WHO classification are used for OKCs [4]. Radiographically, it appears as a well-defined radiolucent lesion with smooth or lobulated margins, a unilocular or multilocular structure, and is most frequently located in the posterior region of the mandible [4]. Epidemiological studies in Mexico have described OKCs as relatively common and aggressive lesions [5,6]. In Chile, in a study of 2944 cases of odontogenic cysts, 421 were diagnosed as OKCs [7]; meanwhile, In Brazil, studies by Schuch et al. [8] and Yamashita et al. [9] reported that out of 261,931 records studied, 2513 were diagnosed as OKCs, most frequently occurring in male patients during the third and fourth decades of life, with a marked predilection for the mandible compared to the maxilla. In North America, a study of 312 cases in the United States reported that the incidence of OKCs is highest between the second and third decades of life, with a mandible–maxilla ratio of 2:1 [10]. A multicenter study of 231 cases found that the clinical management and recurrence of these cysts are the most important factors in the management of this lesion [11]. Systematic reviews have indicated that recurrence rates are related to surgical management and that enucleation is associated with recurrence, which has led to the standardization of treatment protocols to improve long-term prognosis [12]. In Europe, multicenter studies have emphasized the importance of standardizing epidemiological and therapeutic criteria for the management of OKCs [13]. Ahlfors et al. examined 319 OKCs in 255 patients in Sweden, describing clinically significant findings such as a male predilection (ratio of 2:1) and an approximate recurrence rate of 27% [14]. Observational studies in the Portuguese population analyzed the prevalence of these lesions and recommended the need for long-term follow-up and accurate diagnoses [15]. A 36-year study in China reported that the mandibular region was affected in 66.87% of cases, with a recurrence rate of 17.79% [16]. In South Korea, there is a higher frequency in males and higher incidence in the third decade of life [17]. Studies in Southeast Asia (Malaysia and Singapore) and Turkey have confirmed these observations, highlighting the importance of posterior mandibular localization and the need for rigorous clinical follow-up, regardless of the histological variant or the patient’s ethnic background [18,19]. The recurrence rates reported for these cysts are variable, ranging from 15% to 63% [20]. The management of OKCs requires an accurate histopathological diagnosis, detailed imaging evaluation, and long-term postoperative follow-up due to their high recurrence rates. The purpose of the present study is to describe the clinical, imaging, and histopathological characteristics of OKCs in a case series diagnosed in the Mexican population, making an important epidemiological contribution to the region and contributing to a better understanding of the behavior of this pathology.

## 2. Materials and Methods

### 2.1. Ethical Approval

The study protocol was approved by the Research Ethics Committee of the Faculty of Dentistry of the Universidad Autónoma del Estado de México (CEICIEAO-2025-012), and the research was conducted in accordance with the ethical principles established in the Declaration of Helsinki [21].

### 2.2. Study Design and Case Selection

A retrospective, descriptive, cross-sectional, multicenter study design was carried out, which was developed through interinstitutional collaboration among the Faculty of Dentistry of the Universidad Autónoma del Estado de México (UAEMEX), the “Licenciado Adolfo López Mateos” Medical Center, and other private diagnostic centers in Mexico. The research is reported in accordance with the STROBE recommendations for observational research [22,23]. To identify studies for inclusion, a systematic search was performed in the PubMed, Scopus, and Web of Science databases using Boolean operators: (“Odontogenic keratocyst” OR “Keratocystic odontogenic tumor”) AND (epidemiology OR prevalence OR recurrence). The most representative global series were selected to establish a global frame of reference through a comparative analysis. The American continent, considered as a single geographic unit, was subdivided for the purposes of this analysis into the regions of Latin America and North America, allowing for a comparison of OKC behavior in different contexts. The selected series were as follows. i. Latin America: Data were taken from studies with reference cohorts (Yamashita et al. (*n* = 16) [9], França et al. (*n* = 40) [24], Ledesma-Montes et al. (*n* = 304) [5], Mosqueda-Taylor et al. (*n* = 856) [6], Ochsenius et al. (*n* = 421) [7], and Schuch et al. (*n* = 2497) [8]). ii. North America: Classic and contemporary series were used to compare recurrence rates and clinical behavior (Brannon et al. (*n* = 312) [10] and Kinard et al. (*n* = 231) [11]). iii. Asia: Epidemiological reports were included due to their volume and focus on recurrence (Zhao et al. (*n* = 489) [16], Myoung et al. (*n* = 256) [17], and Ngeow et al. (*n* = 61) [18]). iv. Europe: Fundamental series were included for the validation of diagnostic and therapeutic criteria (Ahlfors et al. (*n* = 319) [14] and Boffano et al. (*n* = 415) [13]).

### 2.3. Sample Acquisition

For sample collection, records were retrieved from the files of the oral pathology laboratories and the maxillofacial surgery departments of the participating institutions for the period 2015–2024 (Table S1). To ensure the reliability of multicenter data, specific inclusion, exclusion, and elimination criteria were established.

#### 2.3.1. Inclusion Criteria

a. Patients with a confirmed histopathological diagnosis of OKC based on the criteria established by the WHO tumor classification [4,25]. b. Cases with available H&E-stained slides and paraffin blocks with sufficient histological tissue to prepare new sections if required for re-evaluation. c. Cases with accessible and sufficiently detailed clinical and radiographic records (e.g., patient age, sex, and anatomical location of the lesion).

#### 2.3.2. Exclusion Criteria

a. Cases with incomplete clinical records preventing precise data extraction. b. Archived histological preparations of poor quality that precluded reaching a definitive diagnostic consensus and with insufficient tissue in the paraffin block to prepare new sections. c. Duplicate cases to avoid data overlap from patients who might have been treated or diagnosed at more than one participating center; records were cross-checked using the patient’s initials, age, and the anatomical location of the lesion.

#### 2.3.3. Elimination Criteria

a. Cases where paraffin blocks or slides suffered damage, loss, or irreparable deterioration during the histopathological re-evaluation process. b. Clinical records whose subsequent follow-up revealed that the lesion was part of nevoid basal cell carcinoma syndrome (Gorlin–Goltz syndrome) in order to keep the cohort strictly limited to isolated or non-syndromic keratocysts. c. Patients whose printed or digital clinical records were inaccessible or permanently restricted due to changes in privacy policies or restructuring of archives at the participating centers during the data collection phase.

#### 2.3.4. Cross-Verification Protocol and Quality Control

To ensure the integrity and uniqueness of the sample in this multicenter study, a cross-verification protocol was implemented to exclude duplicate cases. The unique identifiers of each patient (initials, date of birth, sex, and anatomical location of the lesion) were analyzed across all participating databases. If a case matched these identifiers, it was confirmed as the same patient and the redundant record was excluded, retaining only the original record. The quality of the archived data was ensured through a diagnostic calibration process prior to data extraction: two pathologists (V.H.T-R. and R.G-G.) evaluated a pilot sample of 15 slides, achieving a Kappa index of 0.82, which ensured the reliability of their interpretations. A total of 106 records met all criteria and were included in the final cohort.

### 2.4. Data Collection

For each case, clinical, imaging, and histopathological information was systematically reviewed and recorded in a database designed specifically for this study. The evaluation of imaging characteristics and the histopathologic diagnosis were jointly validated by a maxillofacial surgeon and a pathologist. The following variables were collected: age, sex, lesion location, symptoms, association with an impacted tooth, root resorption, syndromic association, histologically observed satellite cyst or island, inflammatory infiltrate, diagnostic method, treatment, and recurrence of the lesion.

### 2.5. Statistical Analysis

The data were analyzed using descriptive statistics. Categorical variables are expressed as frequencies and percentages, while continuous variables are presented as means (standard deviations) or medians (interquartile ranges). For comparison with global series, descriptive frequency analyses were conducted. For inferential analysis, the association between potential risk factors and recurrence was evaluated using Fisher’s exact test or the Chi-square test for categorical variables, and the Mann–Whitney U test for continuous variables (age and lesion size). Likewise, the Kruskal–Wallis test was used to compare continuous variables among multiple categorical groups, and Spearman correlation was used to analyze the relationship between continuous variables. A *p*-value < 0.05 was considered statistically significant. Statistical analyses were performed using the R programming environment (version 4.4.1).

## 3. Results

### 3.1. Sociodemographic and Clinical Characteristics

**Figure 1:**
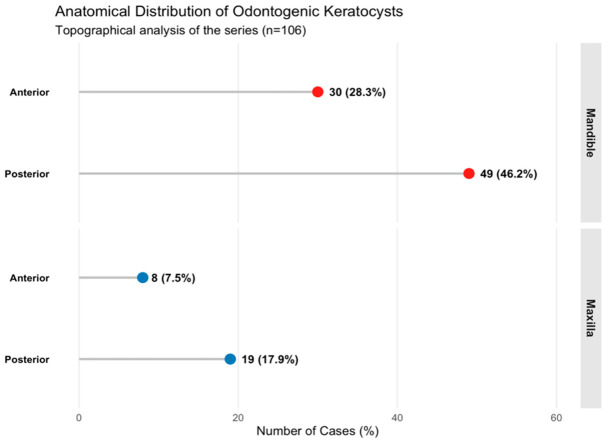
Anatomical distribution of odontogenic keratocyst in the maxilla and mandible within the study cohort (*n* = 106), showing a predilection for the posterior mandibular region. Data are presented as absolute frequencies and percentages (*n*, %).

A total of 106 cases with confirmed diagnosis were included. Patient age ranged from 10 to 86 years, with a mean of 33.4 years (SD: ±17.7). There was a predominance of males, with 61 male patients (57.5%) compared to 45 females (42.5%), demonstrating a male-to-female ratio of 1.36:1. Regarding anatomical location, 8 cases (7.5%) were in the anterior maxilla, 19 cases (17.9%) were in the posterior maxilla (premolar–molar), 30 cases (28.3%) were in the anterior mandible, and 49 cases (46.2%) were in the posterior mandible (premolar–molar). Clinically, 73 patients (68.9%) were asymptomatic, while only 33 patients (31.1%) reported clinical symptoms such as pain or swelling. Table 1 details the sociodemographic, clinical, imaging, and histopathological variables analyzed. Figure 1 shows the anatomical distribution in the maxilla and mandible.

### 3.2. Radiographic Characteristics

**Figure 2:**
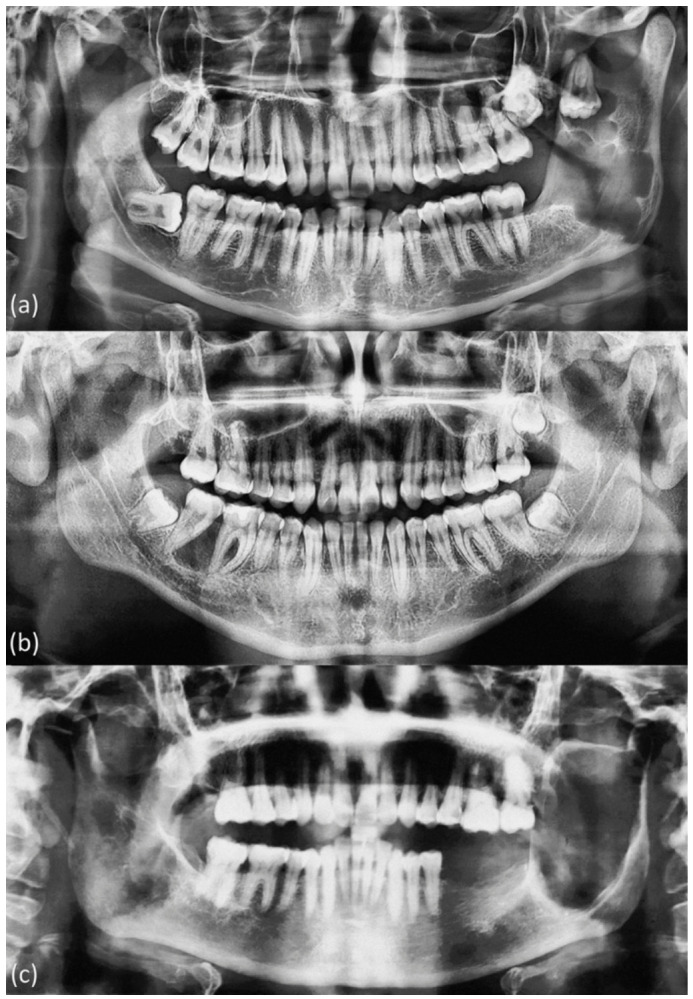
Radiographic images of the odontogenic keratocyst. (**a**) Panoramic radiograph showing a multilocular radiolucent lesion associated with an impacted third molar displaced to the level of the coronoid process, at the angle and ascending ramus of the left mandible. (**b**) Panoramic radiograph showing a unilocular radiolucent lesion at the interradicular and apical level of the right mandibular first and second molars. (**c**) Panoramic radiograph showing a unilocular lesion that was radiographically diagnosed as a residual cyst, extending throughout the entire left ascending mandibular ramus.

An association with impacted teeth was observed in 37 cases (34.9%), whereas no such relationship was noted in the remaining 69 cases (65.1%). Regarding root involvement, resorption was identified in 26 cases (24.5%), while this finding was absent in 80 cases (75.5%). The radiographic features of the OKC, including its varied presentation as uni- or multilocular radiolucent lesions, are shown in Figure 2.

### 3.3. Histopathological and Diagnostic Characteristics

**Figure 3:**
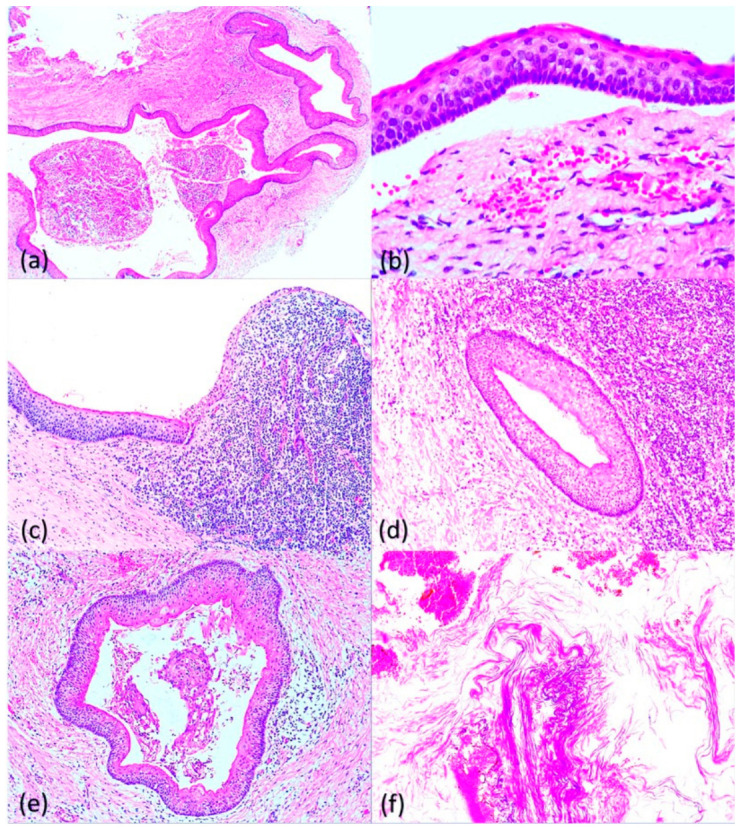
Histological images of odontogenic keratocyst. (**a**) Parakeratinized epithelium, composed of 4 to 8 cell layers with a satellite island (H&E, 100×). (**b**) Cystic epithelium at higher magnification, characteristic of odontogenic keratocyst, basal layer with palisaded hyperchromatic nuclei and a slightly corrugated surface (H&E, 400×). (**c**) Areas of keratocyst epithelium with an inflammatory process morphologically suggestive of an inflammatory cyst (H&E, 100×). (**d**,**e**) Islands of odontogenic keratocyst epithelium invading the capsule, one of them (d) with an inflamed capsule (H&E, 400×). (**f**) Cytological smear from fine-needle aspiration biopsy (FNAB) with abundant structures compatible with keratin strands (H&E, 100×).

From a histopathological perspective, the most common type of epithelial lining observed was parakeratinized squamous epithelium, which was present in the majority of cases. Regarding the inflammatory infiltrate, chronic inflammation was identified in 62 cases (58.5%), while 44 cases (41.5%) showed no inflammatory infiltrate in the subepithelial stroma. Concerning the diagnostic method, 56 (52.8%) were diagnosed via incisional biopsy, 39 (36.8%) via excisional biopsy, 2 (1.9%) via fine-needle aspiration biopsy (FNAB), 6 (5.7%) via FNAB plus incisional biopsy, and 3 (2.8%) via FNAB plus excisional biopsy. Regarding the treatment performed, the most common procedure was curettage with peripheral ostectomy (which included burring of the cavity bone), which was performed in 57 of the 106 cases analyzed (53.8%). Marsupialization was carried out in 21 cases (19.8%), while decompression was performed in 14 cases (13.2%). In 14 cases (13.2%), the type of treatment was not specified in the clinical records. The histopathological features of the odontogenic keratocyst, including epithelial and inflammatory findings, are shown in Figure 3.

### 3.4. Recurrence Analysis

The retrospective evaluation of the records documented recurrence in three cases (2.8%). In 96 cases (90.6%), the records did not report evidence of recurrence in the last clinical note and, in 7 cases (6.6%), the status could not be evaluated due to loss to follow-up. To assess the influence of clinical, sociodemographic, radiographic, histopathological, and diagnostic variables on recurrence, a univariate inferential analysis was performed using the 99 cases with available follow-up information; Fisher’s exact test was used for categorical variables and the Mann–Whitney U test was used for continuous variables. The univariate inferential analysis (Table 2) showed that none of the variables reached statistical significance (*p* > 0.05) as an independent predictor of recurrence. Likewise, comparison of patients with and without recurrence using the Mann–Whitney U test did not reveal significant differences in lesion size (*p* = 0.629) or patient age (*p* = 0.062). Due to limitations in the availability of longitudinal data and the multicenter nature of the study, it was not possible to retrieve the exact duration of follow-up in months for the cohort. Therefore, the documented 2.8% corresponds to a crude recurrence rate calculated from the available records and not to a disease-free survival rate; this limitation, together with the retrospective design of this research, requires caution when interpreting the absence of statistically robust associations.

### 3.5. Analysis of Correlations and Biological Behavior

The relationships between continuous and categorical variables were evaluated using inferential tests. The Spearman correlation between patient age and lesion size did not show a significant relationship (Rho = 0.000; *p* = 1.000; *n* = 106), indicating that, in this series, lesion growth is independent of patient age. Likewise, the Kruskal–Wallis test was used to assess whether patient age varied according to the anatomical location of the lesion. No statistically significant differences were found (Chi^2^ = 0.773; *p* = 0.856), suggesting that the topographic presentation of the odontogenic keratocyst is not influenced by patient age in our population. Table 3 shows the results from the correlation analysis and comparisons of clinical variables.

## 4. Discussion

The literature on odontogenic keratocysts (OKCs) is extensive and diverse in regions such as North America, Europe, and Asia, providing a solid global reference framework. The comparison of the detailed epidemiological data from our series with that of international cohorts is summarized in Table S2, while Figure S1 visually illustrates the geographic distribution of these reference series. Detailed clinicopathological evidence from Mexican and Latin American populations constitutes an essential contribution to international comparative analyses. Our multicenter findings establish a local framework that allows for comparison of the biological behavior of this entity across different population contexts. Figure S1 shows the comparative analysis of these epidemiological data in a global sample of *N* = 5916 cases. Regarding the epidemiological profile, our series revealed an onset age predominantly clustered in young adulthood, a finding consistent with global and regional parameters reported in the literature. This average aligns with cohorts in Latin America, such as the series by Yamashita et al. (mean = 33.19 years) [9] and the Brazilian multicenter series by Schuch et al. [8], which represents the most robust epidemiological reference for the region, as it analyzed 2497 isolated OKC cases with a mean age of 34.2 years. Likewise, the results are in line with recent findings in the Portuguese population, where Monteiro et al. [15] reported a mean age of 36.7 years, consolidating the global predilection of this lesion for young adults. Our data on the age distribution and anatomical location closely correlate with those of the Brazilian series by Schuch et al. [8], confirming that the profile of OKC patients in Mexico is comparable to that reported in other Latin American countries.

The male sex preference observed in our cohort is consistent with previous Mexican literature, matching the findings of Mosqueda-Taylor et al. (58.7% males) [6] and Ledesma-Montes et al. (59.6% males) [5]. The male predominance in OKC cases is a global trend. In North America, Brannon et al. (*N* = 312) [10] reported a male predilection of 56.9%; similar findings were reported by Myoung et al. (*N* = 256; 58.6% males) [17] and Ngeow et al. (*N* = 61; 57.4% males) in Asia [18]. Figure S1, which shows the distribution of these series, offers a comprehensive global perspective. The consistency of these findings reinforces the validity of our clinical observations and highlights the utility of Schuch et al.’s data as a standard of comparison. While our series of 106 cases is smaller in volume than the large multicenter studies, it contributes valuable evidence in the Mexican context, demonstrating that OKC behavior does not show drastic demographic variations across Latin American latitudes, with epidemiological results similar to the previous reports of Mosqueda-Taylor [6] and Ochsenius et al. [7].

In terms of clinical and radiographic features, we identified the mandible as the most common site of involvement, with a marked predilection for the posterior region. This topographic pattern is fully consistent with data from Latin American series such as the study by Ochsenius et al., where 67.5% of lesions were located in the mandible [7], as well as the study by Schuch et al., who confirmed that 77.3% of cases were located in the mandible, mainly in the posterior region [8]. The European multicenter study by Boffano et al. reported a high rate of mandibular involvement (77.1%), corroborating this topographic predilection [13]. In North America, Kinard et al. (*n* = 231) have reported similar location patterns (72.7% mandibular) [11]. Ali et al. highlighted that in the canine region of the maxilla, OKCs can be misdiagnosed as a periapical inflammatory lesion, underscoring the importance of differential diagnosis [26]. The high proportion of asymptomatic patients identified in our study is comparable to that reported in Asian series (Zhao et al., *n* = 489) [16], which associates the indolent behavior of the lesion with advanced stages. Likewise, the association of our lesions with unerupted teeth is a finding that supports the need for rigorous differential diagnosis with dentigerous cysts, a phenomenon described by Brannon et al. [10] and Ali et al. [26].

From a histopathological perspective, our microscopic findings are consistent with the global standard described in recent Latin American reports such as França et al. (*n* = 40) [24] and classical European series (Ahlfors et al., *n* = 319) [14]. In our cohort, the presence of risk structures in the fibrous capsule, such as epithelial islands and satellite cysts, as well as the combination of both alterations, was documented. This histopathological analysis is complemented by the evidence from Schuch et al., who rigorously defined OKCs by their lining of parakeratinized stratified squamous epithelium and emphasize that the precise identification of these microscopic structures is fundamental to unify the diagnostic criteria in multicenter studies [8]. The presence of risk structures in the fibrous capsule, such as epithelial islands and satellite cysts, has historically been associated with lesion aggressiveness in various cohorts. For example, an Asian study reported the presence of satellite cysts in 30.1% of cases (Myoung et al., *n* = 256) [17]. In our series, although these structures were identified in a relevant proportion of cases, no independent statistical association with recurrence was demonstrated. Nevertheless, given their described biological significance, we suggest that their presence be recorded during histopathological evaluation as a factor that could justify closer clinical surveillance, although additional prospective studies are needed to confirm their specific predictive value for recurrence in this population. Regarding treatment and recurrence, the reviewed literature shows variability. Blanas et al. concluded that resection offers the lowest recurrence rate [12]. In contrast, studies such as that by Kinard et al. reported a recurrence rate of 19% following enucleation [11]. Comparing our results, the long-recurrence ratio observed in our study was numerically lower than that reported in large international series (Asia: Zhao et al., 15.27% [16]; North America: Kinard et al., 19% [11]; Europe: Ahlfors et al., 27% [14]). However, this numerical difference should be interpreted with caution. Given the retrospective nature and fragmented follow-up times in our clinical records, this value represents a crude rate and not a disease-free survival rate. It is noteworthy that the Brazilian cohort of França et al. documented a recurrence rate of 45%, highlighting the importance of long-term follow-up [24]. As suggested by Izgi et al. [19] and Al-Moraissi et al. [20], the therapeutic approach should be individualized. The lack of standardized protocols for both surgical management and long-term follow-up remains a significant challenge in OKC research, as described by Boffano et al. [13]. This observation is consistent with the findings of Al-Moraissi et al. [20] and Kinard et al. [11], who note that variability in follow-up periods among different centers biases the reported recurrence rates. Consequently, as observed in our cohort, lower crude recurrence rates may simply reflect early patient dropout or incomplete retrospective follow-up rather than true disease-free survival. This consensus in the recent literature highlights the urgent need to establish global, standardized surveillance guidelines to accurately assess therapeutic outcomes. While achieving global standardization remains a long-term goal, the immediate clinical implications of this study are highly relevant for daily clinical practice. Specifically, our findings underscore the need to maintain a high index of suspicion in the face of radiolucent lesions that do not respond to conventional therapies—such as endodontic treatment—or display unusual radiographic features, as these may represent an OKC. Early recognition and timely referral for biopsy are essential to prevent complications associated with their aggressive growth. Likewise, for clinical specialists, particularly those involved in surgical and endodontic management, our study emphasizes that effective long-term control does not depend solely on the surgical technique employed. It relies crucially on detailed histopathological evaluation, in which the identification of aggressive features—such as satellite cysts and epithelial islands—should directly guide management toward more intensive and individualized postoperative surveillance protocols.

## 5. Study Limitations

This study presents limitations related to its retrospective and multicenter design.

The presence of data labeled as NR in our comparative analysis reflects the heterogeneity of clinical records, an inherent limitation of retrospective studies that has been previously documented in the literature. For example, Ledesma-Montes et al. reported cases with undetermined data (ND) in their analysis, and Mosqueda-Taylor et al. also noted similar limitations in the recording of complete clinical information in their historical series. Our decision to explicitly report these missing data as NR underscores our commitment to methodological transparency, avoiding the extrapolation of unverifiable data.

A main limitation is the loss of longitudinal data over the ten-year period. Cases with incomplete clinical, imaging, or histopathological information were excluded to prioritize diagnostic accuracy and clinicopathological reliability over obtaining a larger number of cases. Consequently, although this strict methodological selection restricted the final sample size to 106 cases, the resulting cohort is representative. As shown in Table S2, this sample provides an epidemiological contribution comparable to that of several international reference series, preserving the descriptive validity of the study both nationally and globally.

However, this decision prevented the performance of survival statistical analyses and, thus, the crude recurrence rate reported may represent an underestimation of the long-term clinical reality. Another methodological weakness lies in the inability to perform a detailed analysis of ancestry or ethnic variables. In this study, origin is recorded by state within the Mexican Republic; however, this information is insufficient to infer the precise genetic composition of the cohort given the marked population mobility and the absence of standardized collection of ethnic identity data in clinical records. This limitation made it impossible to evaluate possible differences in biological aggressiveness or in the prevalence of OKC characteristics in relation to specific subpopulations. Despite the study’s limitations, the robustness of our sample and the histopathological analysis strengthen its descriptive validity. Our results demonstrate that the clinicopathological features in the Mexican population are consistent with those reported in international series, underscoring the urgent need for future national multicenter studies implementing standardized follow-up protocols to optimize the management of this complex pathology.

## 6. Conclusions

This series of 106 cases provides current epidemiological information on odontogenic keratocysts in the Mexican population. Comparing our findings with previous reports in Mexico, such as those by Mosqueda-Taylor and Ledesma-Montes, shows continuity in the demographic and clinical profile of this entity in the country. Likewise, the comparison with series from Latin America, the United States, Europe, and Asia confirmed that the presentation patterns, such as patient age and the anatomical predilection for the posterior mandible, are consistent across different populations, which validates our database within an international reference framework. A central aspect of our analysis was the detailed histopathological evaluation of the capsule. The identification of epithelial islands and satellite cysts provides valuable elements for the biological characterization of the lesion. While these structures are globally recognized as potential indicators of aggressiveness, our findings emphasize the importance of their systematic recording during microscopic diagnosis as a prudent measure for stratifying patient prognosis, pending future research that will clarify their independent role in recurrence risk. Regarding surgical management, there was variability in the techniques employed, ranging from simple enucleation and curettage with peripheral ostectomy to more extensive resections, depending on the clinical criteria. In our series, curettage with peripheral ostectomy was associated with a low recurrence rate. However, due to the retrospective nature of the study and variability in clinical follow-up times, this data should be interpreted with caution; the reported figure represents a crude recurrence rate, not a disease-free survival rate.

This limitation indicates the need to implement prospective and prolonged surveillance protocols to ensure local control of the pathology. Therapeutic success in the treatment of odontogenic keratocysts depends on a multidisciplinary approach. Clinical efficacy is based on the integration of imaging findings with a rigorous histopathological assessment aimed at identifying markers of aggressiveness that allow the surgical technique to be personalized according to the biological profile of each patient, thus ensuring the stability of long-term outcomes.

## Figures and Tables

**Table 1:** Clinicopathological characteristics and follow-up of odontogenic keratocyst cases.

Variable/Clinical Category	Absolute Frequency (*n*)	Percentage (%)
**Demographic Characteristics**		
**Age (years)**		Range: 10–86
Mean ± SD	33.4 ± 17.7	
**Sex**		
Male	61	57.5%
Female	45	42.5%
**Clinical Characteristics**		
**Symptomatology**		
Asymptomatic	73	68.9%
Symptomatic (pain or swelling)	33	31.1%
**Association with impacted tooth**		
Not associated	69	65.1%
Associated	37	34.9%
**Root canal treatment**		
Not registered	105	99.1%
Yes (in 2 dental organs)	1	0.9%
**Imaging Features**		
Anatomical location		
Posterior mandible (premolar–molar)	49	46.2%
Anterior mandible	30	28.3%
Posterior maxilla (premolar–molar)	19	17.9%
Anterior maxilla	8	7.5%
**Root resorption**		
Absent	80	75.5%
Present	26	24.5%
**Histopathological Features**		
Satellite epithelial findings		
None	77	72.6%
Satellite cysts	17	16.0%
Satellite epithelial islands	8	7.5%
Both alterations	4	3.8%
**Inflammatory infiltrate in the subepithelial stroma**		
Chronic inflammation	62	58.5%
Absent	44	41.5%
**Diagnostic method**		
Incisional biopsy	56	52.8%
Excisional biopsy	39	36.8%
FNA + incisional biopsy	6	5.7%
FNA + excisional biopsy	3	2.8%
FNA	2	1.9%
**Treatment performed**		
Curettage with peripheral ostectomy	57	53.8%
Marsupialization	21	19.8%
Decompression	14	13.2%
Not specified	14	13.2%
**Evolution and Follow-up**		
**Evidence of recurrence**		
Absent	96	90.6%
Present	3	2.8%
Not evaluable (lost to follow-up)	7	6.6%

**Table 2:** Analysis of factors associated with recurrence.

Variable	Subgroup	*N*	Recurrence (*n* = 3)	*p*-Value (Fisher)	Odds Ratio (CI 95%)
**Age**	≤28/>28	99		0.097	8.61 (0.433–171) *
**Sex**	Man/Woman	99	3/0	0.255	0.176 (0.008–3.49) *
**Anatomical location**	PM/AM/PMx/AMx	99	2/1/0/0	1.000	N/A **
**Radiography**	Unilocular/Multilocular/NS	99	0/2/1	0.319	N/A **
**Symptomatology**	Asymptomatic/Symptomatic	99	3/0	0.550	0.297 (0.0149–5.93) *
**Association with impacted tooth**	Not associated/Associated	99	2/1	1.000	0.955 (0.083–10.9)
**Root resorption**	Absent/Present	99	1/2	0.168	6.00 (0.52–69.1)
**Root Canal Treatment**	No/Yes	99	3/0	1.000	9.10 (0.31–265) *
**Diagnostic method**	IB/EB/F + IB/F + EB/F	99	0/2/1/0/0	0.091	N/A **
**Epithelium Type**	Para/Ortho	99	3/0	1.000	2.94 (0.13–65.9) *
**Inflammatory infiltrate**	Yes/No	99	3/0	0.255	0.176 (0.008–3.49) *
**Satellite Structure**	Absent/SC/EI/BA	99	2/1/0/0	0.620	N/A **
**Type of surgery**	C + PO/M/D/NS	99	2/0/0/1	0.563	N/A **

Abbreviations: PM = posterior mandible; AM = anterior mandible; PMx = posterior maxilla; AMx = anterior maxilla; IB = incisional biopsy; EB = excisional biopsy; F + IB = FNA + incisional biopsy; F + EB = FNA + excisional biopsy; F = FNA; SC = satellite cyst; EI = epithelial island; BA = both alterations; C + PO = curettage + peripheral ostectomy; M = Marsupialization; D = decompression; NS = not specified. * Odds Ratio (OR) calculated using the Haldane–Anscombe correction due to the presence of null frequencies (cells with zero cases) in the contingency table. ** N/A indicates that the OR could not be calculated or was not applicable due to data distribution.

**Table 3:** Analysis of correlations and group comparisons using non-parametric tests.

Statistical Test	Analyzed Variables	Statistician	*p*-Value
Spearman correlation	Age (years) vs. Lesion size (cm)	0.000 (Rho)	1.000
Kruskal–Wallis test	Age (years) vs. Anatomical location	0.773 (Chi^2^)	0.856

## Data Availability

The data presented in this study are available on request from the corresponding authors. The data are not publicly available due to privacy and ethical restrictions regarding patient clinical histories.

## Supplementary Materials

The following supporting information can be downloaded at https://www.mdpi.com/article/10.3390/diagnostics16142194/s1. Table S1: Distribution of samples by reference center; Table S2: Distribution of clinical and radiographic variables in international series; Figure S1: Comparative distribution of odontogenic keratocyst cases.

## References

[1] Philipsen H.P. (1956). Om keratocyster (kolesteatomer) i kaeberne. Tandlaegebladet.

[2] Barnes L., Eveson J.W., Reichart P., Sidransky D. (2005). World Health Organization Classification of Tumours. Pathology and Genetics of Head and Neck Tumours.

[3] El-Naggar A.K., Chan J.K.C., Grandis J.R., Takata T., Slootweg P.J. (2017). WHO Classification of Head and Neck Tumours.

[4] Bishop J.A., Chan J.K.C., Gale N., Helliwell T., Hyrcza M.D., Lewis J.S. (2024). WHO Classification of Head and Neck Tumours.

[5] Ledesma-Montes C., Hernández-Guerrero J.C., Garcés-Ortíz M. (2000). Clinico-pathologic study of odontogenic cysts in a Mexican sample population. Arch. Med. Res..

[6] Mosqueda-Taylor A., Irigoyen-Camacho M.E., Díaz-Franco M.A., Torres-Tejero M.A. (2002). Quistes odontogénicos. Análisis de 856 casos. Med. Oral.

[7] Ochsenius G., Escobar E., Godoy L., Peñafiel C. (2007). Odontogenic cysts: Analysis of 2944 cases in Chile. Med. Oral Patol. Oral Cir. Bucal.

[8] Schuch L.F., de Arruda J.A.A., Mosconi C., Kirschnick L.B., de Carvalho Pinho R.F., Viveiros S.K., Abreu L.G., do Amaral-Silva G.K., da Silva L.P., Martins-Chaves R.R. (2020). A Brazilian multicentre study of 2497 isolated cases of odontogenic keratocysts. Oral Dis..

[9] Yamashita F.C., Pinto G.N.S., Chicarelli M., Iwaki L.C.V., Tolentino E.S., Iwaki Filho L. (2019). Odontogenic keratocysts: A 22-year epidemiological study and case report. Rev. Fac. Odontol. Porto Alegre.

[10] Brannon R.B. (1976). The odontogenic keratocyst: A clinicopathologic study of 312 cases. Part I. Clinical features. Oral Surg. Oral Med. Oral Pathol..

[11] Kinard B., Hansen G., Newman M., Dennis P., Haeffs T., Perez S., Hamao-Sakamoto A., Steed M., Hughes P., August M. (2019). How well do we manage the odontogenic keratocyst? A multicenter study. Oral Surg. Oral Med. Oral Pathol. Oral Radiol..

[12] Blanas N., Freund B., Schwartz M., Furst I.M. (2000). Systematic review of the treatment and prognosis of the odontogenic keratocyst. Oral Surg. Oral Med. Oral Pathol. Oral Radiol. Endod..

[13] Boffano P., Cavarra F., Agnone A.M., Brucoli M., Ruslin M., Forouzanfar T., Ridwan-Pramana A., Rodríguez-Santamarta T., de Vicente J.C., Starch-Jensen T. (2022). The epidemiology and management of odontogenic keratocysts (OKCs): A European multicenter study. J. Craniomaxillofac. Surg..

[14] Ahlfors E., Larsson A., Sjögren S. (1984). The odontogenic keratocyst: A benign cystic tumor?. J. Oral Maxillofac. Surg..

[15] Monteiro L., Santiago C., do Amaral B., Al-Mossallami A., Albuquerque R., Lopes C. (2021). An observational retrospective study of odontogenic cyst’s and tumours over an 18-year period in a Portuguese population according to the new WHO Head and Neck Tumour classification. Med. Oral Patol. Oral Cir. Bucal.

[16] Zhao Y.F., Wei J.X., Wang S.P. (2002). Treatment of odontogenic keratocysts: A follow-up of 255 Chinese patients. Oral Surg. Oral Med. Oral Pathol. Oral Radiol. Endod..

[17] Myoung H., Hong S.P., Hong S.D., Lee J.I., Lim C.Y., Choung P.H., Lee J.H., Choi J.Y., Seo B.M., Kim M.J. (2001). Odontogenic keratocyst: Review of 256 cases for recurrence and clinicopathologic parameters. Oral Surg. Oral Med. Oral Pathol. Oral Radiol. Endod..

[18] Ngeow W.C., Zain R.B., Yeo J.F., Chai W.L. (2000). Clinicopathologic study of odontogenic keratocysts in Singapore and Malaysia. J. Oral Sci..

[19] Izgi E., Mollaoglu N., Simsek M.B. (2021). Prevalence of odontogenic cysts and tumors on Turkish sample according to latest classification of World Health Organization: A 10-year retrospective study. J. Oral Maxillofac. Pathol..

[20] Al-Moraissi E.A., Kaur A., Gomez R.S., Ellis E. (2023). Effectiveness of different treatments for odontogenic keratocyst: A network meta-analysis. Int. J. Oral Maxillofac. Surg..

[21] World Medical Association (2013). World Medical Association Declaration of Helsinki: Ethical principles for medical research involving human subjects. JAMA.

[22] von Elm E., Altman D.G., Egger M., Pocock S.J., Gøtzsche P.C., Vandenbroucke J.P. (2007). The Strengthening the Reporting of Observational Studies in Epidemiology (STROBE) statement: Guidelines for reporting observational studies. BMJ.

[23] STROBE Initiative (2025). STROBE Checklists (Cohort, Case-Control, and Cross-Sectional Studies). https://www.strobe-statement.org/checklists/.

[24] França J.A., de Sousa S.F., Diniz M.G., Pereira T.S.F., de Resende T.A.C., dos Santos J.N., Gomez R.S., Gomes C.C. (2018). Absence of BRAFV600E mutation in odontogenic keratocysts. J. Oral Pathol. Med..

[25] Soluk-Tekkesin M., Wright J.M. (2022). The World Health Organization classification of odontogenic lesions: Summary of the changes of the 2022 (5th) edition. Turk. J. Pathol..

[26] Ali M., Baughman R.A. (2003). Maxillary odontogenic keratocyst: A common and serious clinical misdiagnosis. J. Am. Dent. Assoc..

