# Supplementary material for: Clinicopathological Characteristics of Odontogenic Keratocysts in the Mexican Population
Source: Diagnostics (Basel). 2026 Jul 14;16(14):2194. doi: 10.3390/diagnostics16142194 (PMC13409477; doi:10.3390/diagnostics16142194)
Supplement: Supplementary file 1 [file diagnostics-16-02194-s001.zip › diagnostics-4381222-supplementary.pdf]

Table S1: Distribution of Samples by Reference Center (Ordered from Least to Greatest)

Laboratory	Reference Center	Cases (n)	Percentage (%)
**Laboratory 1**<br>**(MedOral SF Group, SLP)**	Orocentro Oral Medicine and Pathology Clinic, School of Dentistry, UAEMex, Toluca, Mexico. School of Stomatology, Universidad Autónoma de San Luis Potosí, SLP, Mexico.	6	5.7%
**Laboratory 2**<br>**(Private Practice, CDMX)**	Division of Health Sciences, School of Dentistry, Universidad Intercontinental, Mexico City, Mexico.	9	8.5%
**Laboratory 3**<br>**(CDMX / Puebla)**	Interdisciplinary Health Sciences Center (CICS), Santo Tomás Unit (UST), IPN, Mexico City, Mexico.<br>School of Dentistry, Universidad Nacional Autónoma de México, Mexico City, Mexico.<br>Centro Maxilofacial Puebla, Puebla, Mexico. Hospital General de Cholula, San Andrés Cholula, Puebla, Mexico.	11	10.4%
**Laboratory 4**<br>**(Oral Pathology Mexico, Toluca))**	School of Dentistry, UAEMex, Toluca, Mexico.<br>Orocentro Oral Medicine and Pathology Clinic, School of Dentistry, UAEMex, Toluca, Mexico.<br>Maxillofacial Surgery Service, "Licenciado Adolfo López Mateos" Medical Center, Toluca, Mexico.<br>Pediatric Dentistry, School of Dentistry, UAEMex, Toluca, Mexico.	35	33.0%
**Laboratory 5**<br>**(Durango)**	School of Dentistry, UABC, Mexicali, Mexico.<br>Academic Unit of Dentistry, Universidad Autónoma de Guerrero, Acapulco, Mexico.<br>General Hospital of Acapulco, Secretaría de Salud Guerrero, Acapulco, Mexico. General Hospital La Perla, Nezahualcóyotl, Estado de México, Mexico.Specialty in Oral Surgery and Comprehensive Diagnosis and Oral Pathology, Private Practice, Mexico.<br>Academic Unit of Dentistry, Universidad Autónoma de Zacatecas, Zacatecas, Mexico.<br>Research Department, School of Dentistry, UJED, Durango, Mexico.	45	42.5%
**Total**		**106**	**100.0%**

Table S2. Distribution of clinical and radiographic variables in the study series.

Author, year	Country	N (OKC)	Men, n (%)	Women, n (%)	Mandible, n (%)	Maxilla, n (%)	Recurrence
Latin America							
Rios-Orta et al. (2026)	Mexico	106	61 (57.5%)	45 (42.5%)	79 (74.5%)	27 (25.5%)	2.8%
Schuch et al (2020) [8].	Brazil	2,497	1,308 (52.4%)	1,189 (47.6%)	1,930 (77.3%)	567 (22.7%)	NR
Ledesma-Montes et al (2000) [5].	Mexico	57	34 (59.6%)	21 (36.8%)	NR	NR	NR
Mosqueda-Taylor et al (2002) [6].	Mexico	184	108 (58.7%)	76 (41.3%)	NR	NR	NR
Yamashita et al (2019) [9].	Brazil	16	8 (50.0%)	8 (50.0%)	11 (68.7%)	5 (31.3%)	12.5%
França et al (2021) [24].	Brazil	40	17 (42.5%)	23 (57.5%)	35 (87.5%)	5 (12.5%)	45.0%
Ochsenius et al (2007) [7].	Chile	421	231 (54.9%)	190 (45.1%)	284 (67.5%)	137 (32.5%)	NR
Norteamérica							
Brannon (1976) [10]	U.S.	312	161 (56.9%)	119 (42.0%)	191 (65.4%)	101 (34.6%)	12.0%
Kinard et al (2019) [11].	U.S.	231	125 (54.1%)	106 (45.9%)	168 (72.7%)	63 (27.3%)	19.0%
Europa							
Ahlfors et al (1984) [14].	Sweden	319	167 (65.0%)	88 (35.0%)	~75.0%	~25.0%	27.0%
Boffano et al (2022) [13].	Europe	415	249 (61.0%)	156 (39.0%)	320 (77.1%)	95 (22.9%)	14.4%
Monteiro et al (2021) [15].	Portugal	50	15 (42.9%)	20 (57.1%)	42 (84.0%)	8 (16.0%)	24.0%
Asia							
Zhao et al (2002) [16]	China	489	319 (65.9%)	165 (34.1%)	327 (66.9%)	162 (33.1%)	12.2%
Myoung et al (2001) [17].	South Korea	256	150 (58.6%)	106 (41.4%)	196 (76.5%)	60 (23.5%)	58.3%
Ngeow et al (2000) [18].	Singapore/Malaysia	61	35 (57.4%)	26 (42.6%)	40 (65.5%)	19 (31.0%)	NR
Izgi et al (2021) [19].	Turkey	64	35 (54.7%)	29 (45.3%)	51 (79.7%)	13 (20.3%)	NR

NR = Not Reported. The NR designation indicates data that were not specified in the original literature. This variability is a known limitation in retrospective multicenter studies due to the lack of standardization

in historical clinical records. We have chosen to report these values as "NR" instead of performing arbitrary imputations in order to preserve the integrity of the original data. The data from Schuch et al. (2020) correspond to consolidated results from their multicenter population series. N (Regional sample): Refers to the sum of cases reported in the bibliographic series selected by region: Latin America (Schuch et al., Mosqueda-Taylor et al., Ledesma-Montes et al., França et al., Yamashita et al., Ochsenius et al.); North America (Brannon, Kinard et al.); Europe (Ahlfors et al., Boffano et al., Monteiro et al.); Asia (Zhao et al., Myoung et al., Ngeow et al.).

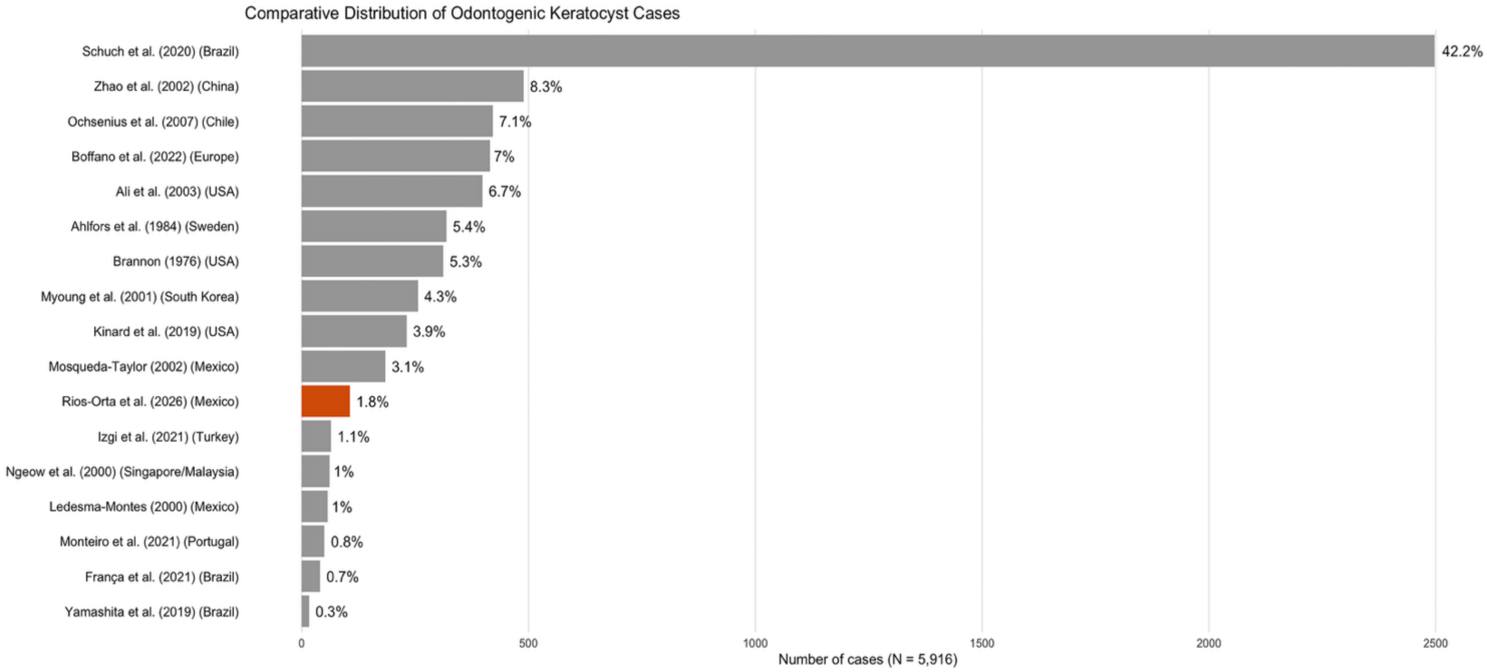

Figure S1. Distribution map of the epidemiological series analyzed: international reference framework for contrasting the clinicopathological profile of the odontogenic keratocyst. The geographic regions of the cohorts (Latin America, North America, Europe, and Asia) that support the global comparison of the results are shown. Original figure designed on the BioRender platform, which provides us the relevant licenses for scientific publication.
